# Delirium and in-hospital falls in older adults: factors associated with fall occurrence and number of falls

**DOI:** 10.1007/s41999-026-01482-2

**Published:** 2026-04-20

**Authors:** Justine Falciola, Christophe Graf, Aline Mendes

**Affiliations:** 1 Division of Geriatrics and Rehabilitation, Department of Rehabilitation and Geriatrics, University Hospitals of Geneva and University of Geneva, Chemin du Pont-Bochet 3, 1226 Thônex, Geneva Switzerland; 2 Cognitive and Behavioural Geriatric Unit, Division of Geriatrics and Rehabilitation, University Hospitals of Geneva and University of Geneva, Geneva, Switzerland

**Keywords:** Delirium, In-hospital falls, Fall count, Acute geriatric care, Functional status, Psychotropic medications

## Abstract

**Purpose:**

To examine the association between delirium and in-hospital falls in older adults, including fall occurrence and number of falls, and to assess whether fall-related factors differ according to delirium status.

**Methods:**

We conducted a retrospective cohort study of patients aged ≥ 65 years hospitalized in acute geriatric units between 2022 and 2024. Delirium was identified using ICD-10 codes, Confusion Assessment Method (CAM) documentation, and physician-entered diagnoses. Fall occurrence (≥ 1 fall) was analysed using multivariable logistic regression and number of falls using negative binomial regression. Analyses were stratified according to delirium status.

**Results:**

Among 12,866 hospitalizations, 1751 patients (13.6%) experienced at least one in-hospital fall. Delirium occurred in 2889 patients (22.5%) and was independently associated with fall occurrence (OR 1.51, 95% CI 1.32–1.72). Male sex (OR 1.56, 95% CI 1.39–1.74), Parkinson’s disease (OR 1.52, 95% CI 1.23–1.89), and antipsychotic exposure were also associated with falls. Baseline functional status showed a significant interaction with delirium status (p = 0.0002): higher functional independence was associated with lower fall risk in patients without delirium (OR per 10-point FIM increase 0.91, 95% CI 0.89–0.94), but not in those with delirium (OR 0.99, 95% CI 0.95–1.03). Delirium was also independently associated with a higher fall count (IRR 1.47, 95% CI 1.31–1.66).

**Conclusion:**

Delirium is independently associated with both the occurrence and number of in-hospital falls. Once delirium develops, fall risk appears to reflect a state of acute neurocognitive instability in which baseline functional status loses much of its discriminative value for risk stratification.

**Supplementary Information:**

The online version contains supplementary material available at https://doi.org/10.1007/s41999-026-01482-2.

## Key Summary Points

**Aim:**

To determine whether delirium is associated with both the occurrence and number of in-hospital falls in older adults and whether delirium modifies the structure of fall-related factors.

**Findings:**

In a large cohort of hospitalized older adults, delirium was independently associated with both fall occurrence and number of falls, indicating sustained vulnerability throughout hospitalization. While core structural factors such as male sex, Parkinson’s disease, and antipsychotic exposure remained associated with falls regardless of delirium status, baseline functional status lost its discriminative value once delirium developed.

**Message:**

Delirium represents a persistent risk state for in-hospital falls in which traditional functional risk stratification appears less informative, underscoring the need for delirium-focused and sustained fall prevention strategies.

**Supplementary Information:**

The online version contains supplementary material available at https://doi.org/10.1007/s41999-026-01482-2.

## Introduction

In-hospital falls are a frequent adverse event among older adults, with reported rates ranging from 3 to 14 falls per 1000 bed-days in acute care settings, and are associated with increased morbidity, functional decline, prolonged hospitalisation, and mortality [1–3]. Delirium affects up to 20–30% of hospitalised older adults and is characterised by acute disturbances in attention, cognition, and psychomotor behaviour [4–8]. These features plausibly increase vulnerability to falls by impairing hazard awareness, judgement, and postural control [9]. Several observational studies have reported an association between delirium and in-hospital falls, with patients with delirium exhibiting up to a twofold increased risk compared with non-delirium patients [10]. However, most studies have focused exclusively on the overall occurrence of a fall and have examined fall risk factors in heterogeneous inpatient populations, without evaluating whether delirium modifies the relative contribution of established fall-related determinants [11, 12].

Data on repeated in-hospital falls in the context of delirium are particularly limited. Recurrent falls may reflect a sustained vulnerability state rather than a single precipitating event and are associated with worse clinical outcomes [13, 14]. Patients with delirium may be especially susceptible to repeated falls due to persistent or fluctuating cognitive and behavioural disturbances throughout hospitalisation. Whether delirium is associated not only with fall occurrence but also with fall count per patient during hospitalization, and whether predictors such as baseline functional status and acute medical conditions retain their discriminative value once delirium develops, remains largely unexplored [15, 16].

The objectives of this study were to examine the association between delirium and (i) the occurrence of in-hospital falls and (ii) number of falls in older adults hospitalised in acute geriatric units. We further aimed to compare factors associated with falls according to delirium status. We hypothesised that delirium would be independently associated with both fall occurrence and fall count, and that the presence of delirium would attenuate the discriminative value of baseline functional status and acute medical characteristics for fall risk.

## Methods

### Design, setting, and population

We conducted a retrospective observational cohort study at the Trois-Chêne Hospital, the specialized geriatric centre of the University Hospitals of Geneva. The hospital provides approximately 200 acute geriatric beds and admits most patients through the emergency department (92%). All consecutive hospitalizations of patients aged 65 years and older admitted to acute geriatric units between January 1, 2022, and December 31, 2024, were eligible for inclusion. In accordance with Swiss legislation on research involving human subjects, the requirement for informed consent was waived due to the retrospective observational design of the study and the use of routinely collected, de-identified data. The study was approved by the Geneva Cantonal Research Ethics Commission (Project-ID 2024-00010).

### Data collection

Data were extracted from the hospital’s electronic medical records and administrative databases. Collected variables included demographic characteristics, baseline functional and cognitive status, clinical diagnoses, laboratory parameters at admission, use of medical devices, and exposure to psychotropic medications during hospitalization.

Baseline functional status was assessed using the Functional Independence Measure (FIM), a validated 18-item scale ranging from 18 to 126, with lower scores indicating greater functional dependence [17]. Pain intensity at admission was measured using the Visual Analogue Scale (VAS) ranging from 0 (no pain) to 10 (worst imaginable pain) [18]. Laboratory variables collected at admission included C-reactive protein, and leukocyte count, analysed as continuous variables.

Clinically relevant acute diagnoses and chronic comorbidities were identified using ICD-10 diagnostic codes and grouped into predefined clinical categories, including infectious, metabolic, neurological, and cardiovascular conditions (Supp Material). Use of medical devices during hospitalization, including urinary catheterization and oxygen supplementation, was recorded as binary variables. Exposure to psychotropic medications was captured as binary indicators for each molecule (benzodiazepines, antipsychotics, and non-benzodiazepine hypnotics).

Delirium was defined as delirium present at hospital admission, identified from clinical documentation in the emergency department or at the time of admission to the acute geriatric ward. Delirium cases were identified using ICD-10 diagnostic codes, explicit documentation of delirium in the clinical problem list, or Confusion Assessment Method (CAM) assessments when available, with possible overlap between these sources. In this cohort, 81.4% of delirium cases were identified through ICD coding, 63.5% through problem-list documentation, and 7.3% through CAM assessments. Although CAM screening is recommended in routine clinical practice, its systematic recording in structured electronic documentation was not fully integrated during the study period, and CAM assessments were therefore inconsistently captured in the dataset.

The primary outcome was the occurrence of at least one in-hospital fall, defined as any unintentional descent to the ground or to a lower level occurring during hospitalization and recorded in the institutional incident reporting system. The secondary outcome was number of falls, defined as the total number of falls per patient and modelled using count-based regression methods. In the study setting, all fall events are systematically documented in the medical record and the institutional incident reporting system, irrespective of injury severity or clinical consequences, including non-injurious falls.

### Statistical analysis

Baseline characteristics were summarized for the whole cohort and compared between patients with and without at least one in-hospital fall. Continuous variables were described using means and standard deviations and compared using Student’s t tests. Categorical variables were reported as counts and percentages and compared using χ^2^ tests. Two-sided p values < 0.05 were considered statistically significant.

The association between delirium and the occurrence of at least one in-hospital fall was examined using multivariable logistic regression models. Results are presented as odds ratios (ORs) with 95% confidence intervals (CIs). Delirium was included as the main exposure variable. Models were adjusted a priori for demographic characteristics, baseline functional status, pain intensity at admission, cognitive diagnoses, comorbidities, acute medical conditions, laboratory values at admission, use of medical devices, and exposure to psychotropic medications during hospitalization. Age, FIM score, pain intensity, laboratory values, and leukocyte count were entered as continuous variables. Psychotropic medications were modelled as binary indicators for each molecule.

To explore whether factors associated with falls differed according to delirium status, stratified multivariable logistic regression analyses were performed separately in patients with and without delirium, using the same covariate structure as in the main model, except for delirium status itself.

The number of falls per patient during hospitalization was analysed using negative binomial regression models to account for overdispersion of count data. Incidence rate ratios (IRRs) with 95% CIs were reported. The same covariates used in the logistic regression models were included. As for fall occurrence, stratified negative binomial models were fitted according to delirium status to assess consistency of associations across strata. Overdispersion was assessed and supported the use of negative binomial models. Because some patients contributed more than one hospital stay, analyses were conducted at the stay level and standard errors were clustered at the patient level to account for potential non-independence of observations across repeated admissions. Statistical analyses were performed using Stata version 17 (StataCorp, College Station, TX, USA).

## Results

### Characteristics of the study population

Among the 12,866 hospitalizations included in the study, 1751 patients (13.6%) experienced at least one in-hospital fall. Delirium was present in 2889 patients (22.5%) and was more frequent among fallers than non-fallers (40.5% vs 19.6%, p < 0.001). Compared with non-fallers, patients who fell were more frequently male (49.8% vs 38.6%, p < 0.001) and had significantly longer hospital stays (mean 22.6 ± 14.6 vs 15.4 ± 10.3 days, p < 0.001).

Baseline functional status was lower in fallers than in non-fallers (mean FIM score 64.8 ± 26.1 vs 77.4 ± 26.7, p < 0.001), whereas age did not differ between groups (mean 84.8 ± 7.3 vs 84.8 ± 7.2 years, p = 0.90). Pain intensity at admission was slightly lower among fallers (mean VAS 2.5 ± 2.6 vs 2.7 ± 2.5, p < 0.001).

Fallers more frequently presented dementia (21.9% vs 11.9%, p < 0.001), Parkinson’s (7.9% vs 4.5%, p < 0.001), and stroke (1.3% vs 0.7%, p = 0.009). They also had a higher prevalence of acute medical conditions, including pneumonia (33.3% vs 25.9%, p < 0.001), metabolic disturbances (63.8% vs 48.1%, p < 0.001), neurological factors (16.4% vs 10.9%, p < 0.001), acute heart failure (32.2% vs 27.1%, p < 0.001), and acute kidney failure (27.1% vs 21.4%, p < 0.001). Recent hip fracture was also more frequent among fallers (1.5% vs 0.2%, p < 0.001).

Exposure to psychotropic medications during hospitalization was markedly more frequent among fallers. Antipsychotic use was substantially higher in fallers, including haloperidol (20.8% vs 5.7%), quetiapine (34.1% vs 12.5%), and risperidone (4.7% vs 1.7%) (all p < 0.001). Benzodiazepine exposure was also more common among fallers, notably lorazepam (25.8% vs 14.4%) and oxazepam (12.5% vs 9.1%), as was midazolam use (4.8% vs 2.0%) (all p < 0.001). In contrast, zolpidem exposure did not differ significantly between groups. Use of medical devices was more frequent among fallers, including urinary catheterization (36.9% vs 21.7%, p < 0.001) and oxygen supplementation (31.5% vs 26.7%, p < 0.001). Detailed characteristics are presented in Table 1.

**Table 1:** Characteristics of fallers and non-fallers

	Non-fallers	Fallers	Total	Test
N	11,115 (86.4%)	1751 (13.6%)	12,866 (100.0%)	
Female sex n (%)	6827 (61.4%)	879 (50.2%)	7706 (59.9%)	< 0.001
Age y (SD)	84.8 (7.2)	84.8 (7.3)	84.8 (7.2)	0.896
Delirium n (%)	2180 (19.6%)	709 (40.5%)	2889 (22.5%)	< 0.001
LOS d (SD)	15.4 (10.3)	22.6 (14.6)	16.4 (11.2)	< 0.001
VAS for pain 0–10 (SD)	2.7 (2.5)	2.5 (2.6)	2.7 (2.5)	< 0.001
FIM 13–126 (SD)	77.4 (26.7)	64.8 (26.1)	75.7 (27.0)	< 0.001
Dementia n (%)	1326 (11.9%)	383 (21.9%)	1709 (13.3%)	< 0.001
Mild Cognitive Impairment n (%)	191 (1.7%)	34 (1.9%)	225 (1.7%)	0.508
Stroke n (%)	75 (0.7%)	22 (1.3%)	97 (0.8%)	0.009
Parkinson n (%)	501 (4.5%)	139 (7.9%)	640 (5.0%)	< 0.001
Sepsis n (%)	299 (2.7%)	57 (3.3%)	356 (2.8%)	0.180
Pneumonia n (%)	2883 (25.9%)	583 (33.3%)	3466 (26.9%)	< 0.001
Psychiatric factors n (%)	2060 (18.5%)	444 (25.4%)	2504 (19.5%)	< 0.001
Neurological factors n (%)	1207 (10.9%)	287 (16.4%)	1494 (11.6%)	< 0.001
Other infections n (%)	3390 (30.5%)	793 (45.3%)	4183 (32.5%)	< 0.001
Metabolic factors n (%)	5342 (48.1%)	1118 (63.8%)	6460 (50.2%)	< 0.001
Recent hip fracture n (%)	23 (0.2%)	27 (1.5%)	50 (0.4%)	< 0.001
Factors associated with Hypoxia n (%)	997 (9.0%)	153 (8.7%)	1150 (8.9%)	0.751
Comorbidities n (%)	7119 (64.0%)	1220 (69.7%)	8339 (64.8%)	< 0.001
Inflammatory/auto-immune diseases n (%)	123 (1.1%)	20 (1.1%)	143 (1.1%)	0.895
Acute liver failure n (%)	11 (0.1%)	4 (0.2%)	15 (0.1%)	0.140
Acute respiratory failure n (%)	962 (8.7%)	158 (9.0%)	1120 (8.7%)	0.611
Acute pulmonary embolism n (%)	249 (2.2%)	55 (3.1%)	304 (2.4%)	0.021
Acute heart failure n (%)	3007 (27.1%)	564 (32.2%)	3571 (27.8%)	< 0.001
Acute kidney failure n (%)	2382 (21.4%)	475 (27.1%)	2857 (22.2%)	< 0.001
Alprazolam	286 (2.6%)	55 (3.1%)	341 (2.7%)	0.169
Haloperidol	638 (5.7%)	364 (20.8%)	1,002 (7.8%)	< 0.001
Lorazepam	1,602 (14.4%)	451 (25.8%)	2,053 (16.0%)	< 0.001
Midazolam	225 (2.0%)	84 (4.8%)	309 (2.4%)	< 0.001
Olanzapine	72 (0.6%)	24 (1.4%)	96 (0.7%)	0.001
Oxazepam	1,008 (9.1%)	218 (12.5%)	1,226 (9.5%)	< 0.001
Quetiapine	1,391 (12.5%)	597 (34.1%)	1,988 (15.5%)	< 0.001
Risperidone	194 (1.7%)	82 (4.7%)	276 (2.1%)	< 0.001
Zolpidem	730 (6.6%)	128 (7.3%)	858 (6.7%)	0.247
CRP mean (SD)	49.6 (62.4)	47.2 (60.2)	49.2 (62.1)	0.133
Leucocytes mean (SD)	10.0 (5.6)	9.9 (5.6)	10.0 (5.6)	0.463
O2 supplementation n (%)	2964 (26.7%)	551 (31.5%)	3515 (27.3%)	< 0.001
Urinary catheterization n (%)	2408 (21.7%)	646 (36.9%)	3054 (23.7%)	< 0.001

*LOS* length of stay, *VAS* for pain Visual Analogical Scale for pain, *FIM* Functional Independence Measure, *MCI* Mild Cognitive Impairment, *CRP* C-reactive protein

### Delirium and the occurrence of in-hospital falls

In multivariable logistic regression analysis including the whole cohort, delirium during hospitalization was independently associated with an increased risk of in-hospital falls (OR 1.51, 95% CI 1.32–1.72). Male sex (OR 1.56, 95% CI 1.39–1.74), lower baseline functional status (OR per 10-point increase FIM 0.94, 95% CI 0.92–0.96), Parkinson’s disease (OR 1.52, 95% CI 1.23–1.89), and recent hip fracture (OR 4.68, 95% CI 2.44–8.95) were also independently associated with falls.

Exposure to antipsychotic medications was associated with an increased risk of falls, particularly haloperidol (OR 1.86, 95% CI 1.56–2.21), quetiapine (OR 1.97, 95% CI 1.71–2.28), lorazepam (OR 1.32, 95% CI 1.15–1.51) and risperidone (OR 1.63, 95% CI 1.22–2.18). Other psychotropic agents showed weaker or inconsistent associations (Table 2).

**Table 2:** Logistic regression models of factors associated with in-hospital falls

	(1)	(2)	(3)	(4)
	Whole cohort	Whole cohort	Delirium subgroup	No-delirium subgroup
Delirium	2.79***	1.51***		
	[2.51,3.10]	[1.32,1.72]		
Male sex		1.56***	1.55***	1.59***
		[1.39,1.74]	[1.29,1.88]	[1.38,1.83]
Age		1.00	1.00	1.00
		[0.99,1.01]	[0.99,1.01]	[0.99,1.01]
VAS for pain		1.01	1.01	1.01
		[0.98,1.03]	[0.97,1.05]	[0.98,1.04]
FIM per 10-point increase		0.94***	0.99	0.91***
		[0.92,0.96]	[0.95,1.03]	[0.89,0.94]
Alprazolam		1.06	1.14	1.06
		[0.76,1.48]	[0.65,1.99]	[0.70,1.60]
Haloperidol		1.86***	1.77***	2.06***
		[1.56,2.21]	[1.42,2.20]	[1.55,2.73]
Lorazepam		1.32***	1.30*	1.33**
		[1.15,1.51]	[1.05,1.62]	[1.11,1.59]
Midazolam		1.03	1.22	0.85
		[0.77,1.38]	[0.83,1.80]	[0.53,1.36]
Olanzapine		1.27	1.38	1.16
		[0.75,2.16]	[0.66,2.90]	[0.54,2.51]
Oxazepam		1.09	0.90	1.20
		[0.91,1.30]	[0.67,1.21]	[0.96,1.49]
Quetiapine		1.97***	1.86***	2.05***
		[1.71,2.26]	[1.52,2.27]	[1.69,2.49]
Risperidone		1.63***	2.32***	1.10
		[1.22,2.18]	[1.54,3.48]	[0.69,1.74]
Zolpidem		1.19	0.96	1.31*
		[0.96,1.47]	[0.64,1.43]	[1.02,1.68]
Dementia		1.15	1.10	1.14
		[0.98,1.34]	[0.88,1.37]	[0.92,1.42]
MCI		1.12	1.01	1.21
		[0.75,1.67]	[0.53,1.93]	[0.72,2.01]
Stroke		1.10	0.97	1.32
		[0.64,1.91]	[0.45,2.06]	[0.63,2.80]
Parkinson		1.52***	1.61**	1.47**
		[1.23,1.89]	[1.13,2.29]	[1.11,1.93]
Sepsis		0.69*	0.47**	0.85
		[0.50,0.96]	[0.28,0.80]	[0.56,1.27]
Pneumonia		1.17*	1.18	1.18*
		[1.03,1.33]	[0.95,1.46]	[1.00,1.38]
Psychiatric factors		1.25**	1.31*	1.23*
		[1.09,1.43]	[1.05,1.64]	[1.03,1.46]
Neurological factors		1.31***	1.15	1.43***
		[1.12,1.54]	[0.88,1.51]	[1.17,1.74]
Infections		1.43***	1.68***	1.31***
		[1.27,1.61]	[1.38,2.05]	[1.13,1.52]
Metabolic factors		1.47***	1.42***	1.50***
		[1.31,1.65]	[1.17,1.73]	[1.30,1.73]
Recent hip fracture		4.68***	3.43**	5.66***
		[2.44,8.95]	[1.41,8.32]	[2.39,13.41]
Factors associated with hypoxia		0.90	0.97	0.87
		[0.73,1.10]	[0.67,1.39]	[0.68,1.12]
Comorbidities		1.21**	1.21	1.20*
		[1.07,1.36]	[0.99,1.47]	[1.03,1.40]
Inflammatory/auto-immune diseases		1.21	0.94	1.38
		[0.73,2.01]	[0.37,2.40]	[0.76,2.50]
Acute liver failure		1.17	2.88	1.00
		[0.34,3.97]	[0.59,13.91]	[1.00,1.00]
Acute respiratory failure		0.77*	0.89	0.72*
		[0.63,0.95]	[0.62,1.27]	[0.55,0.92]
Acute pulmonary embolism		1.14	1.46	1.03
		[0.83,1.58]	[0.87,2.46]	[0.67,1.59]
Acute heart failure		1.27***	1.05	1.38***
		[1.12,1.44]	[0.83,1.33]	[1.19,1.61]
Acute kidney failure		1.09	1.10	1.08
		[0.96,1.24]	[0.88,1.36]	[0.92,1.27]
CRP		1.00*	1.00	1.00
		[1.00,1.00]	[1.00,1.00]	[1.00,1.00]
Leucocytes		0.99	0.99	0.99
		[0.98,1.00]	[0.97,1.02]	[0.98,1.00]
O_2_ supplementation		1.02	1.14	0.97
		[0.89,1.18]	[0.89,1.46]	[0.82,1.16]
Urinary catheterization		1.17*	1.07	1.23*
		[1.03,1.34]	[0.87,1.33]	[1.04,1.46]
Observations	12,866	12,866	2889	9970

Exponentiated coefficients; 95% confidence intervals in brackets

*VAS for pain* visual analogical scale for pain, *FIM* functional independence measure, *MCI* mild cognitive impairment. *CRP* C-reactive protein

*p < 0.05, **p < 0.01, ***p < 0.001

Stratified analyses according to delirium status showed substantial overlap in factors associated with falls. Male sex (OR 1.55, 95% CI 1.29–1.88 in delirium; OR 1.59, 95% CI 1.38–1.83 in no delirium), Parkinson’s disease (OR 1.61, 95% CI 1.13–2.29 and OR 1.47, 95% CI 1.11–1.93), and recent hip fracture (OR 3.43, 95% CI 1.41–8.32 and OR 5.66, 95% CI 2.39–13.41) remained associated with falls in both groups.

**Fig. 1:**
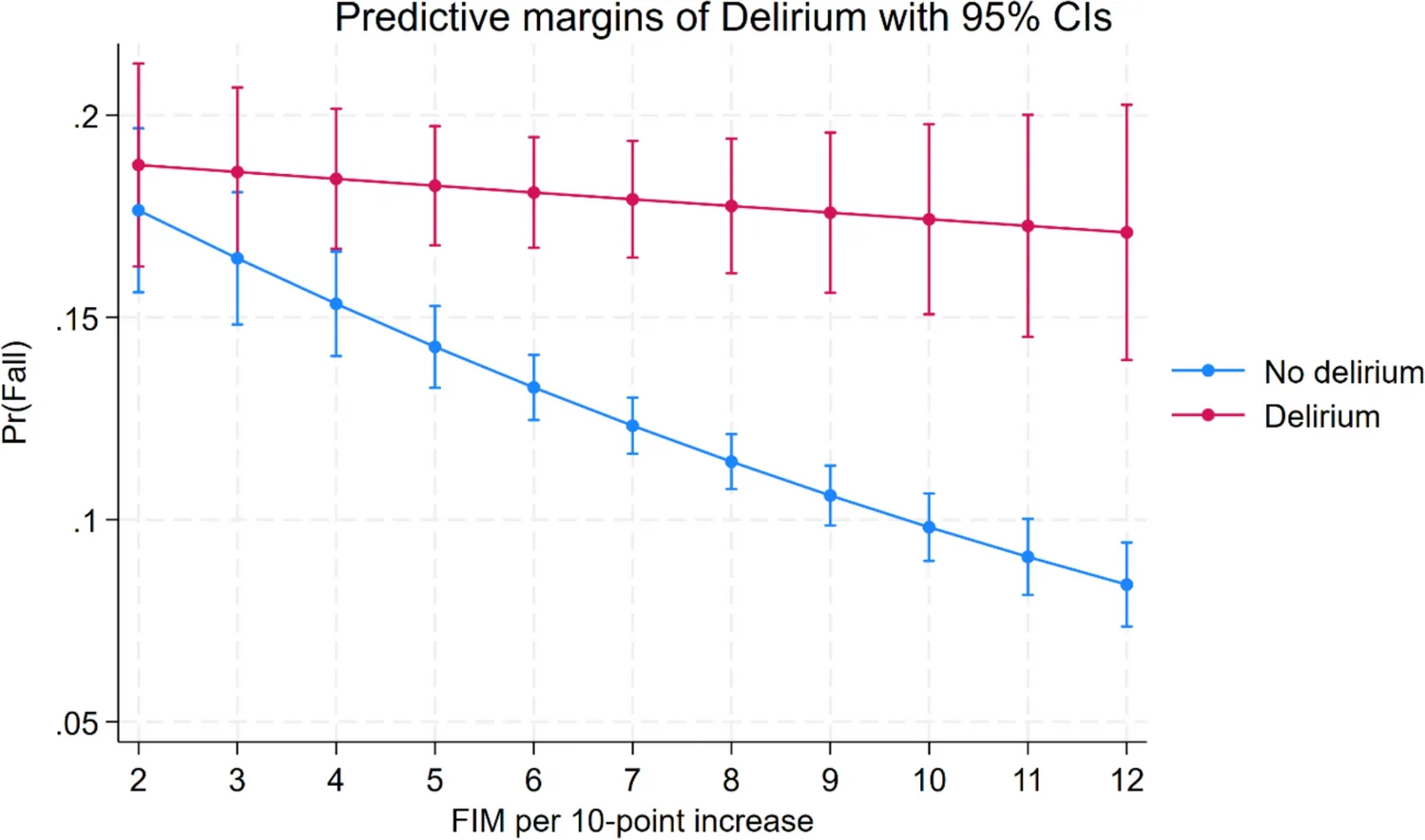
Predicted probability of falls by functional status (FIM) and delirium status. Predictive margins derived from the multivariable regression model showing the probability of falls across levels of functional status (FIM score, expressed in 10-point increments). Error bars represent 95% confidence intervals. *FIM* functional independence measure

Functional status measured by the Functional Independence Measure (FIM) was associated with fall risk among patients without delirium. For each 10-point increase in FIM score, the odds of falling decreased (OR 0.91, 95% CI 0.89–0.94). In contrast, this association was no longer observed among patients with delirium (OR 0.99, 95% CI 0.95–1.03). The delirium × FIM interaction was statistically significant (p = 0.0002), indicating that the predictive value of functional status differed according to delirium status. Marginal effects analysis showed that higher FIM scores were associated with lower fall probability in patients without delirium (risk difference − 0.009 per 10 FIM points, p < 0.001) but not in those with delirium (− 0.002, p = 0.51) (Fig. 1).

### Delirium and number of falls

**Fig. 2:**
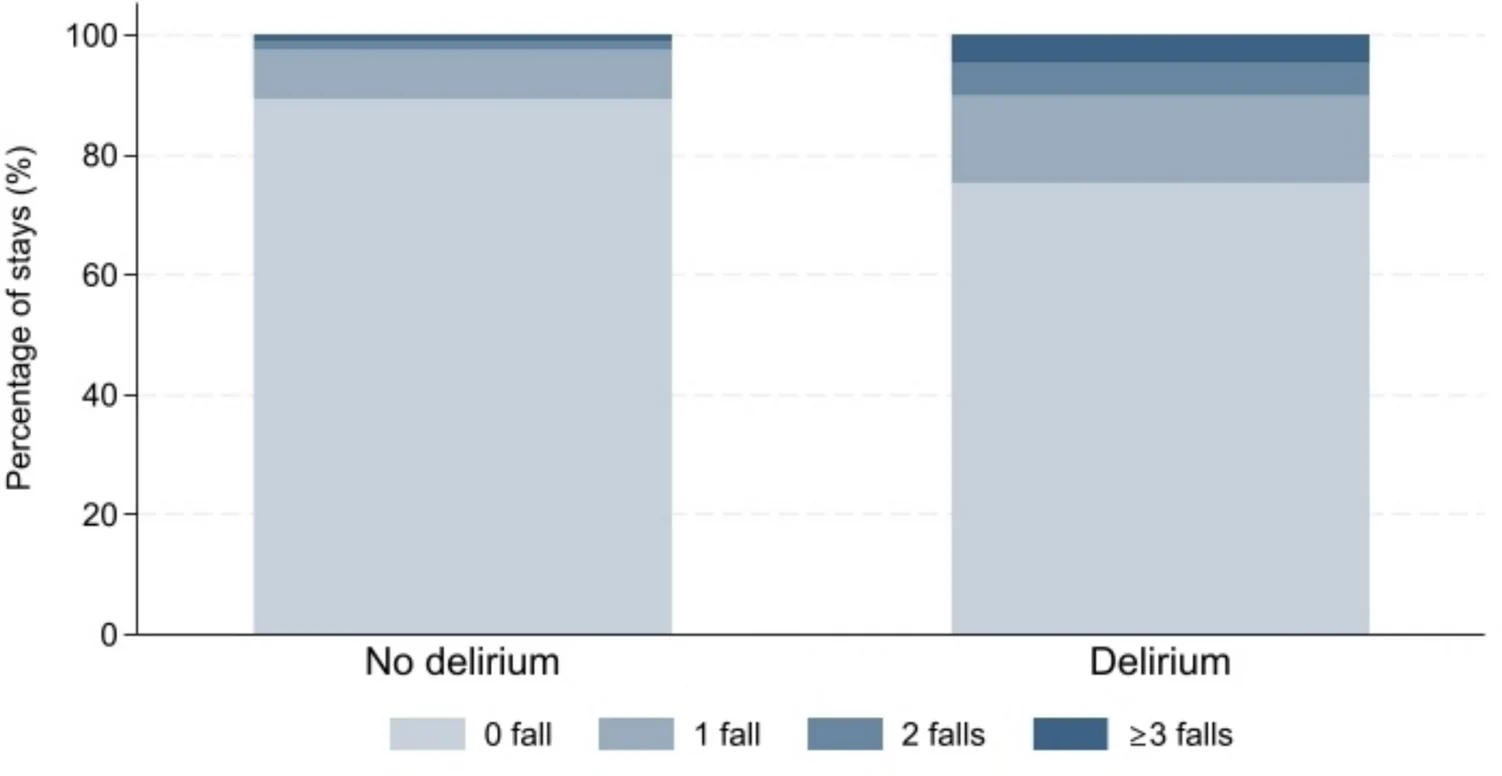
Distribution of falls per stay by delirium status

**Fig. 3:**
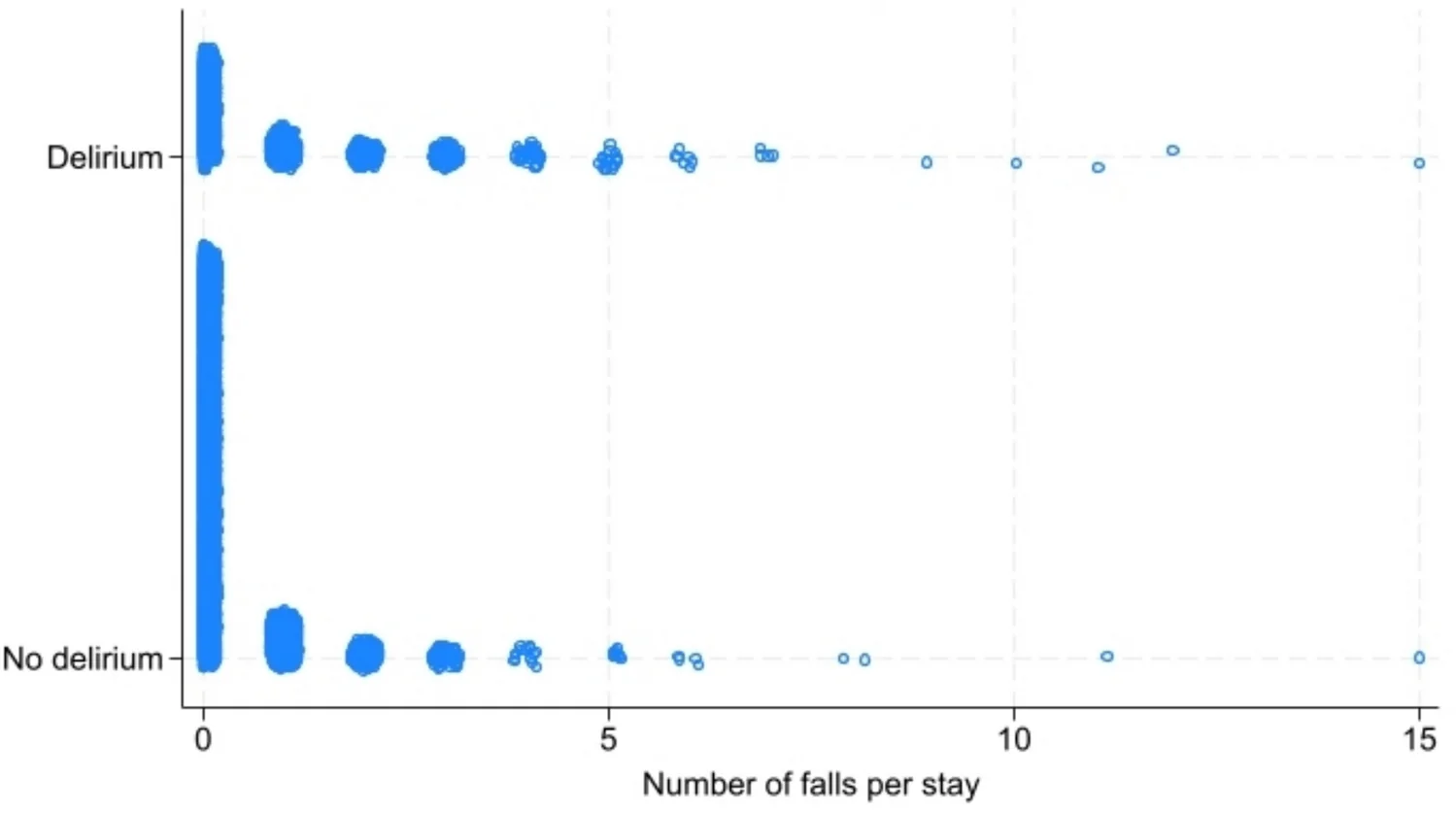
Number of falls per stay by delirium status

The distribution of falls per hospital stay differed markedly according to delirium status, with a higher proportion of multiple falls among patients with delirium (Fig. 2). Patients with delirium also experienced a higher number of falls per stay, consistent with the increased rate of falls observed in multivariable analyses (Fig. 3).

In multivariable negative binomial regression including the whole cohort, delirium was independently associated with an increased rate of falls (IRR 1.47, 95% CI 1.31–1.66). Male sex (IRR 1.56, 95% CI 1.40–1.73), and lower baseline functional status (IRR per 10-point increase FIM 0.94, 95% CI 0.92–0.96) were also associated with a higher number of falls. Parkinson’s disease (IRR 1.46, 95% CI 1.19–1.78) and antipsychotic exposure, particularly haloperidol (IRR 1.79, 95% CI 1.54–2.07) and quetiapine (IRR 1.78, 95% CI 1.57–2.02), showed strong associations with the number of falls (Table 3).

**Table 3:** Negative binomial models of factors associated with number of in-hospital falls

	(1)	(2)	(4)	(5)
	Whole cohort	Whole cohort	Delirium subgroup	No-delirium subgroup
Delirium	2.56***	1.47***		
	[2.31,2.84]	[1.31,1.66]		
Male sex		1.56***	1.48***	1.61***
		[1.40,1.73]	[1.26,1.73]	[1.40,1.84]
Age		1.00	1.00	1.01
		[1.00,1.01]	[0.98,1.01]	[1.00,1.02]
VAS for pain		1.00	1.00	1.00
		[0.98,1.02]	[0.96,1.03]	[0.97,1.03]
FIM per 10-point increase		0.94***	0.97	0.92***
		[0.92,0.96]	[0.94,1.00]	[0.90,0.95]
Alprazolam		1.07	0.98	1.17
		[0.79,1.46]	[0.63,1.53]	[0.79,1.74]
Haloperidol		1.79***	1.65***	2.08***
		[1.54,2.07]	[1.38,1.98]	[1.63,2.66]
Lorazepam		1.15*	1.13	1.17
		[1.01,1.30]	[0.94,1.34]	[0.98,1.39]
Midazolam		0.91	1.08	0.71
		[0.72,1.15]	[0.82,1.44]	[0.47,1.09]
Olanzapine		1.20	1.42	1.06
		[0.79,1.82]	[0.81,2.47]	[0.56,2.03]
Oxazepam		1.00	0.93	1.05
		[0.85,1.17]	[0.72,1.20]	[0.86,1.28]
Quetiapine		1.78***	1.67***	1.89***
		[1.57,2.02]	[1.41,1.97]	[1.57,2.28]
Risperidone		1.61***	2.07***	1.11
		[1.26,2.06]	[1.49,2.88]	[0.74,1.65]
Zolpidem		1.03	0.82	1.11
		[0.85,1.24]	[0.58,1.16]	[0.89,1.38]
Dementia		1.20*	1.04	1.28*
		[1.04,1.38]	[0.86,1.25]	[1.04,1.58]
MCI		1.24	1.10	1.36
		[0.87,1.77]	[0.67,1.79]	[0.84,2.21]
Stroke		1.17	1.14	1.12
		[0.61,2.25]	[0.43,3.01]	[0.57,2.20]
Parkinson		1.46***	1.46**	1.46**
		[1.19,1.78]	[1.10,1.93]	[1.11,1.92]
Sepsis		0.66**	0.49**	0.76
		[0.49,0.90]	[0.31,0.77]	[0.52,1.11]
Pneumonia		1.08	1.06	1.09
		[0.96,1.21]	[0.88,1.27]	[0.93,1.26]
Psychiatric factors		1.26***	1.25*	1.27**
		[1.11,1.43]	[1.03,1.52]	[1.07,1.50]
Neurological factors		1.36***	1.30*	1.43***
		[1.18,1.56]	[1.04,1.63]	[1.19,1.71]
Infections		1.15*	1.26**	1.09
		[1.03,1.29]	[1.06,1.49]	[0.94,1.27]
Metabolic factors		1.16**	1.21*	1.14
		[1.04,1.29]	[1.02,1.43]	[0.99,1.31]
Recent hip fracture		2.26***	1.30	3.21***
		[1.49,3.45]	[0.82,2.05]	[1.98,5.20]
Factors associated with hypoxia		0.92	0.91	0.93
		[0.76,1.11]	[0.69,1.21]	[0.73,1.19]
Comorbidities		1.24***	1.16	1.30***
		[1.11,1.40]	[0.97,1.38]	[1.12,1.51]
Inflammatory/auto-immune diseases		1.08	0.56	1.30
		[0.69,1.70]	[0.25,1.25]	[0.77,2.20]
Acute liver failure		0.86	1.66	0.00***
		[0.44,1.70]	[0.89,3.08]	[0.00,0.00]
Acute respiratory failure		0.73**	0.83	0.68**
		[0.60,0.89]	[0.62,1.13]	[0.53,0.88]
Acute pulmonary embolism		0.91	1.09	0.86
		[0.69,1.19]	[0.76,1.56]	[0.58,1.27]
Acute heart failure		1.10	0.94	1.17*
		[0.98,1.25]	[0.76,1.17]	[1.01,1.36]
Acute kidney failure		1.03	1.01	1.04
		[0.91,1.16]	[0.84,1.21]	[0.89,1.21]
CRP		1.00	1.00*	1.00
		[1.00,1.00]	[1.00,1.00]	[1.00,1.00]
Leucocytes		1.00	1.00	1.00
		[0.99,1.01]	[0.98,1.02]	[0.98,1.01]
O_2_ supplementation		0.97	1.06	0.94
		[0.85,1.11]	[0.87,1.30]	[0.80,1.12]
Urinary catheterization		0.99	0.89	1.05
		[0.88,1.12]	[0.74,1.06]	[0.89,1.24]
Observations	12,866	12,866	2889	9977

Exponentiated coefficients; 95% confidence intervals in brackets

*VAS for pain* visual analogical scale for pain, *FIM* functional independence measure. *MCI* mild cognitive impairment, *CRP* C-reactive protein

*p < 0.05, **p < 0.01, ***p < 0.001

Stratified analyses showed similar patterns across delirium status. Male sex and was consistently associated with a higher number of falls in both delirious and non-delirious patients. Parkinson’s disease remained associated with the number of falls in both strata, as did antipsychotic exposure, including haloperidol (IRR 1.65, 95% CI 1.38–1.98 in delirium; IRR 2.08, 95% CI 1.63–2.66 in no delirium) and quetiapine (IRR 1.67, 95% CI 1.41–1.97 and IRR 1.89, 95% CI 1.57–2.28, respectively).

Baseline functional status remained inversely associated with the number of falls among patients without delirium (IRR per 10-point increase FIM 0.92, 95% CI 0.90–0.95) but not among those with delirium (IRR 0.97, 95% CI 0.94–1.00). Dementia was not independently associated with the number of falls after stratification.

## Discussion

In this large cohort of older adults hospitalized in acute geriatric wards, delirium was independently associated with both the occurrence and the number of in-hospital falls. Beyond this overall association, our findings show that delirium modifies the structure of fall-related risk factors. While several core determinants such as male sex and Parkinson’s disease remained consistently associated with falls regardless of delirium status, the predictive value of baseline functional status was markedly attenuated once delirium developed.

In patients with delirium, falls occurred across a broader clinical spectrum, potentially including individuals who would otherwise be considered at lower risk based on functional or medical characteristics [19]. Moreover, the stronger association observed between delirium and fall count during hospitalization compared with overall falls supports the notion that delirium confers a sustained vulnerability to repeated events during hospitalization [20, 21].

Previous studies have identified delirium as a risk factor for in-hospital falls [9, 12]. A large study of over 29,000 admissions in routine clinical practice found that patients who screened positive for delirium during their hospital stay had significantly increased adjusted odds of falling as inpatients [10]. Furthermore, studies have reported associations with male sex, neurological disease, and psychotropic exposure in mixed inpatient populations [22–24]. However, these studies rarely examined whether such associations persist specifically among patients with delirium or how delirium alters the relative contribution of established fall-related risk factors [25]. In this context, our stratified analyses extend existing knowledge by showing that while core structural predictors remain relevant in delirious patients, baseline functional status and acute medical characteristics lose discriminative value once delirium develops.

This finding suggests that delirium may introduce mechanisms that override or flatten the protective gradient conferred by better functional performance at admission [26]. Delirium is characterized by acute disturbances in attention, executive function, perception, and psychomotor regulation, which directly impair hazard awareness, postural control, and the ability to adapt movements to environmental constraints [27, 28]. Even patients with higher baseline functional independence and physical performance may therefore become unable to appropriately judge risk, follow safety instructions, or seek assistance, placing them at increased risk of unanticipated mobility attempts. In this context, preserved strength or mobility may paradoxically facilitate falls by enabling impulsive or poorly coordinated movements in the setting of attentional and executive dysfunction [29, 30]. Moreover, delirium is frequently accompanied by fluctuating levels of arousal, psychomotor agitation, or disinhibition, further increasing the likelihood of unsafe ambulation irrespective of premorbid functional capacity. Consequently, once delirium develops, fall risk therefore appears to reflect a state of neurocognitive instability rather than baseline physical capacity. Functional status, which remains an important determinant of fall risk in non-delirious patients, becomes less informative for risk stratification when delirium is present.

The attenuation of associations between acute medical characteristics once delirium develops can be explained by two complementary mechanisms. First, delirium may serve as a more proximal marker of acute physiological stress with respect to fall risk than its individual precipitating diagnoses. While diverse acute medical conditions (e.g., infection, metabolic disturbances, organ failure) contribute variably to illness severity, the development of delirium signals that systemic stress has impacted central nervous system function and compromised cognitive control of behaviour. Once this state arises, the specific nature or severity of underlying diagnoses appears less discriminative for fall risk, consistent with the notion that delirium integrates multiple pathways of physiological insult into a unified neurocognitive vulnerability. Second, the weakened associations may also reflect differences in exposure to fall opportunities across clinical presentations. Patients with severe acute medical illness who remain largely bedridden, require continuous monitoring, or are tethered to supportive devices may have reduced unassisted mobility and therefore fewer opportunities to fall.

The association between delirium and number of in-hospital falls provides further insight into the nature of fall risk once delirium develops. While most previous studies have focused on the occurrence of one fall, our findings indicate that delirium is consistently associated with number of falls, suggesting a sustained and cumulative vulnerability rather than a single precipitating event. Importantly, the pattern of factors associated with number of falls closely mirrored that observed for first falls. To our knowledge, few studies have quantified the association between delirium and number of in-hospital falls using count-based models, highlighting delirium as a key driver not only of fall onset but also of repeated fall events during hospitalization.

Exposure to antipsychotic medications was associated with higher odds of falls. However, this association should be interpreted cautiously. In the context of delirium, psychotropic prescribing often reflects the severity of neuropsychiatric symptoms, such as agitation or behavioral disturbances, that themselves increase fall risk, raising the possibility of confounding by indication. Because information on dose, timing of initiation, and symptom severity was not available, causal inference cannot be established. Psychotropic exposure should therefore be interpreted primarily as a marker of underlying clinical vulnerability rather than a direct determinant of falls [31, 32].

These findings support delirium as a key target for inpatient fall prevention [33]. A systematic review and trial sequential meta-analysis have shown that delirium prevention interventions are associated with a significant reduction in in-hospital falls among older adults, indicating that addressing delirium can directly translate into fall risk reduction [16, 34]. Our results extend this evidence by showing that delirium is associated not only with fall occurrence but also with number of in-hospital falls per patient, reinforcing the need for early delirium detection [35] and sustained implementation of non-pharmacological delirium prevention measures as a synergic component of fall prevention strategies in acute geriatric care [36–39].

This study is based on a large cohort of older adults hospitalized in acute geriatric units, enabling robust multivariable and stratified analyses. Delirium was identified using a multimodal approach reflecting routine clinical practice, enhancing external validity. A key strength is the assessment of fall recurrence using count-based models, providing novel insight into cumulative fall risk during hospitalization. The inclusion of functional, cognitive, clinical, and medication-related variables allowed a comprehensive evaluation of fall-related risk structures.

Limitations include the retrospective observational design, which precludes causal inference and allows for residual confounding. Delirium identification relied on clinical documentation and diagnostic coding and may have underestimated the prevalence of hypoactive delirium. Falls may have been underreported despite the use of clinical and institutional incident reporting systems. Because precise time stamps for delirium onset were not available, delirium could not be modeled as a time-varying exposure, and it cannot be completely excluded that some delirium diagnoses were documented after a fall. Similarly, because the timing of psychotropic medication initiation was not available, it cannot be fully determined whether some prescriptions occurred after the fall, and the observed associations may partly reflect confounding by indication. Medication exposure was not exhaustively assessed for all drug classes known to influence fall risk; in particular, antidepressants and antihypertensive agents were not included in the analyses, potentially leading to residual confounding by unmeasured pharmacological factors. In addition, psychotropic exposure was assessed without information on dose or timing. Finally, the single-center geriatric setting may limit generalizability.

## Conclusion

Delirium is independently associated with both the occurrence and the number of in-hospital falls in older adults. Our findings further show that delirium modifies the relationship between baseline functional status and fall risk, attenuating the protective gradient typically associated with higher functional independence. Our findings highlight delirium as a driver of sustained vulnerability throughout hospitalization, supporting delirium-focused and continuous fall prevention strategies in acute geriatric care.

## Supplementary Information

Below is the link to the electronic supplementary material.

Supplementary file1 (DOCX 22 kb)

## Data Availability

De-identified participant data and the study protocol are available upon reasonable request to the corresponding author (aline.mendes@hug.ch) for qualified researchers, subject to institutional and ethical approvals in accordance with Swiss data protection regulations.
